# The relationship between transcription initiation RNAs and CCCTC-binding factor (CTCF) localization

**DOI:** 10.1186/1756-8935-4-13

**Published:** 2011-08-03

**Authors:** Ryan J Taft, Peter G Hawkins, John S Mattick, Kevin V Morris

**Affiliations:** 1 Institute for Molecular Bioscience, University of Queensland, St Lucia, Queensland 4072, Australia; 2 Department of Molecular and Experimental Medicine, The Kellogg School of Science and Technology, The Scripps Research Institute, La Jolla, CA 92037, USA; 3 The Kellogg School of Science and Technology, The Scripps Research Institute, La Jolla, CA 92037, USA

## Abstract

**Background:**

Transcription initiation RNAs (tiRNAs) are nuclear localized 18 nucleotide RNAs derived from sequences immediately downstream of RNA polymerase II (RNAPII) transcription start sites. Previous reports have shown that tiRNAs are intimately correlated with gene expression, RNA polymerase II binding and behaviors, and epigenetic marks associated with transcription initiation, but not elongation.

**Results:**

In the present work, we show that tiRNAs are commonly found at genomic CCCTC-binding factor (CTCF) binding sites in human and mouse, and that CTCF sites that colocalize with RNAPII are highly enriched for tiRNAs. To directly investigate the relationship between tiRNAs and CTCF we examined tiRNAs originating near the intronic CTCF binding site in the human tumor suppressor gene, *p21 *(cyclin-dependent kinase inhibitor 1A gene, also known as *CDKN1A*). Inhibition of CTCF-proximal tiRNAs resulted in increased CTCF localization and increased *p21 *expression, while overexpression of CTCF-proximal tiRNA mimics decreased CTCF localization and *p21 *expression. We also found that tiRNA-regulated CTCF binding influences the levels of trimethylated H3K27 at the alternate upstream *p21 *promoter, and affects the levels of alternate *p21 *(*p21^alt^*) transcripts. Extending these studies to another randomly selected locus with conserved CTCF binding we found that depletion of tiRNA alters nucleosome density proximal to sites of tiRNA biogenesis.

**Conclusions:**

Taken together, these data suggest that tiRNAs modulate local epigenetic structure, which in turn regulates CTCF localization.

## Background

In addition to mRNAs, it is now clear that most eukaryotic genic loci generate a complex network of overlapping short (<200) and long non-protein coding RNA (ncRNA) species [1-3]. This growing catalog of ncRNAs includes a host of small RNA (sRNA) transcripts proximal to transcription start sites (TSSs) [4], some of which are capped [5] or associate with polycomb repressive complex (PCR2) components [6,7]. We have recently described a set of nuclear localized dominantly 18 nucleotide tiny RNAs that are generated from regions immediately downstream of TSSs and conserved across metazoa but absent in fungi and plants [8,9]. We have previously suggested that these transcription initiation RNAs (tiRNAs) may be connected to epigenetic regulation in light of the fact that (i) they originate from the same position relative to the +1 nucleosome in evolutionarily distant animals, suggesting that expression of tiRNAs is influenced by the position of the nucleosome, or vice versa, (ii) they are intimately connected with peaks of RNA polymerase II (RNAPII) binding and levels of gene expression, and (iii) they are enriched genome-wide at chromatin marks associated with the activation, but not elongation, of transcription [4,8,9]. We have also observed that tiRNAs are enriched at sites of CCCTC-binding factor (CTCF) [9], an enigmatic epigenetic regulator that has been recently dubbed 'the master weaver' of the genome [10].

CTCF is a highly conserved zinc finger protein associated with a diverse set of phenomena including epigenetic insulation, imprinting and transcriptional regulation [10-13]. Intriguingly, CTCF has been shown to both positively and negatively regulate gene expression in a gene-specific and context-specific manner [10,14]. A resolution to this apparent incongruity has recently been proposed: CTCF does not directly influence the surrounding genes or transcriptional machinery, but rather acts as a three-dimensional orchestrator of chromatin architecture [10]. CTCF's involvement in a wide range of epigenetic phenomena appears to be the secondary, but undoubtedly regulated, effects of its ability to form specific intrachromosomal and interchromosomal connections [10].

CTCF has been shown to regulate the expression of several tumor suppressor genes, including *p21 *(informal gene name for the cyclin-dependent kinase inhibitor 1A gene, *CDKN1A*) [11,15] and *p16 *(INK4a), the latter by insulating the promoter from silent-state histone modifications such as H3K27 trimethylation (H3K27me3) [16]. CTCF has also recently been shown to be involved in the epigenetic regulation of *frataxin *(*FXN*), a gene mutated and silenced in Friedreich ataxia, which causes progressive damage to the nervous system [17]. Loss of CTCF binding in the 5' untranslated region (UTR) of *FXN *leads to a deficiency of the *FXN *transcript, an increase in *FXN antisense transcript 1*, and heterochromatin formation involving the +1 nucleosome [17]. Given that tiRNAs and RNAPII are intimately connected, and that there is increasing evidence that CTCF and RNAPII are coupled together (see below) [18-22], we speculated that tiRNAs at CTCF-binding sites might be involved in the alteration of local chromatin states, and therefore transcript expression, via indirect regulation CTCF.

## Results and discussion

We have previously shown that tiRNAs isolated from THP-1 cells (a human monocytic leukemia cell line) are systematically enriched at white blood cell CTCF binding sites [9]. To examine if this relationship is preserved across cell and tissue types, and multiple species, we interrogated small RNA enrichments at CTCF binding sites in MCF-7 breast cancer cells and mouse embryonic stem cells (mESCs) (for a full list of data sources please see (Additional file 1, Table S1).

**Figure 1:**
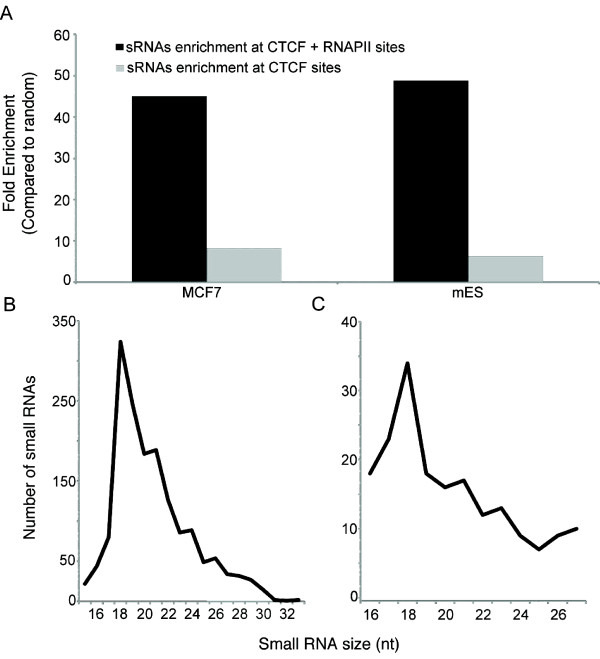
**Enrichment of transcription initiation (ti)RNAs at CCCTC-binding factor-RNA polymerase II (CTCF-RNAPII) sites in human MCF-7 and mouse embryonic stem (mES) cells**. **(a) **Enrichment of tiRNAs at CTCF sites in MCF-7 and mES cells compared to 1,000 simulated random distributions. **(b, c) **Size distribution of tiRNAs that intersect CTCF-RNAPII sites in MCF-7 and mES cells.

Consistent with prior work we found that tiRNAs derived from both MCF-7 and mESCs are enriched approximately sixfold at CTCF binding sites that sit outside TSSs or other annotated genomic features (see Methods and Figure 1a), and show the characteristic 18 nucleotide tiRNA peak (Figure 1b, c). When CTCF binding sites were further refined to include only sites coincident with RNA polymerase II binding (CTCF-RNAPII sites), tiRNA enrichments increased considerably, to approximately 45-fold. Indeed, more than 50% and 20% of MCF-7 and mESC CTCF-RNAPII sites intersect with tiRNAs, respectively (Tables 1 and 2). This relationship appears to bridge the reports indicating that tiRNA biogenesis is a direct result of RNAPII backtracking and nascent transcript cleavage [4,8,9], and recent studies showing that CTCF is directly involved in RNAPII function. Indeed, it has now become clear that (i) a subpopulation of CTCF directly interacts with the large subunit of RNAPII through it's phosphorylated C-terminal tail [21,22], (ii) that in some cases a single CTCF site is both necessary and sufficient to drive RNAPII transcription in the absence of canonical promoters by recruitment of RNAPII [21,22], and (iii) that CTCF specificity for, and regulation of, transcriptionally competent complexes also extends to RNA polymerase I [18-20].

**Table 1:** MCF-7 CCCTC-binding factor (CTCF), RNA polymerase II (RNAPII) and CTCF-RNAPII site intersections with small RNAs (sRNAs)

	Total number of MCF-7 sites	Number that overlap with sRNAs (%)	Total number of sRNAs overlapped (percentage total sRNAs)
RNAPII	10,821	1,165 (11)	2,333 (2)
CTCF	23,857	2,047 (9)	4,731 (4)
CTCF + RNAPII	936	468 (50)	1,609 (1.6)

**Table 2:** Mouse embryonic stem (mES) CCCTC-binding factor (CTCF), RNA polymerase II (RNAPII) and CTCF-RNAPII site intersections with small RNAs (sRNAs)

	Total number of mES sites	Number that overlap with sRNAs (%)	Total number of sRNAs overlapped (percentage total sRNAs)
RNAPII	8,421	575 (7)	1,775 (3)
CTCF	13,469	589 (4)	981 (1.5)
CTCF + RNAPII	434	86 (20)	186 (0.3)

To examine if the association between tiRNAs, CTCF and RNAPII extends beyond MCF-7 and mESCs we identified CTCF sites conserved across an additional eight human cell lines (GM12878, HepG2, HMEC, HSMM, HUVEC, K562, NHEK, and NHLF cells) [23,24] and RNAPII sites conserved across three (K562, GM12878, and HUVEC), and intersected them with nuclear and cytoplasmic small RNAs (sRNAs) from THP-1 and 5-8f cells (a nasopharyngeal carcinoma cell line [25]) and MCF-7 total sRNAs. Despite the fact that these datasets are derived from disparate origins, nuclear sRNAs from THP-1 and 5-8f are 33-fold and 16-fold enriched, respectively, and total sRNAs from MCF cells are 31-fold enriched at conserved CTCF-RNAPII sites (Additional file 2, Figure S1a). Additionally, like the MCF-7 and mESC datasets discussed above, the small RNAs that overlap CTCF-RNAPII sites are dominantly 18 nucleotides, indicating they are tiRNAs (Additional file 2, Figure S1b-d). Overall, greater than 10% of the conserved CTCF sites, and 60% of conserved CTCF-RNAPII sites, overlap with sequences that generate tiRNAs (Additional file 3, Table S2). To further ensure that the tiRNA enrichment at CTCF-RNAPII sites was robust we parsed the conserved human CTCF sites into two groups by origin, 'cancer' and 'normal', and removed all CTCF sites that overlapped with TSSs, repeat masker annotations and small RNAs. Using the most robust RNAPII datasets in each group (MCF-7 and HUVEC for 'cancer' and 'normal', respectively), we found that this dramatically reduced set still shows robust enrichment for tiRNAs at CTCF-RNAPII sites (Additional file 4, Figure S2).

**Figure 2:**
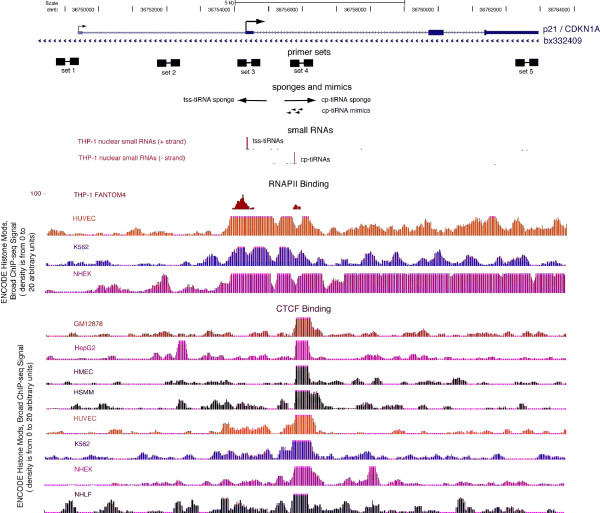
**Schematic depicting the *p21 *(cyclin-dependent kinase inhibitor 1A gene, also known as *CDKN1A*) locus**. The blue lines, boxes and arrows at the top of the image show the p21 and antisense transcripts. Small black boxes connected by black lines depict the five primer pairs used in this study. THP-1 small RNAs (sRNAs) are shown as a density plot in red, followed below by RNA polymerase II (RNAPII) binding from multiple cell types, and CCCTC-binding factor (CTCF) binding across eight cell types. The THP-1 nuclear sRNA deep sequencing data is the deepest sRNA dataset currently available and was therefore, given the conservation of the CTCF site, was used as the basis for the sponge and mimic constructs in all cell lines.

To experimentally interrogate the tiRNA-CTCF-RNAPII relationship we queried for sites in clinically relevant genes and identified a CTCF-RNAPII site with tiRNAs in the first intron of *p21*, which is conserved across both multiple human cell types (Figure 2) and mammalian species (Additional file 5, Figure S3). CDKN1A/p21 is a significant tumor suppressor that acts at the G1 checkpoint to inhibit cell cycle progression [26-29], and its downregulation (but not mutation) is a common feature of many cancers [30-34]. In addition to *p21 *mRNA, the *p21 *locus encodes a number of other transcripts, including alternative p21 transcripts (*p21^alt^*) that originate from a unique promoter located approximately 2 kb upstream of the canonical *p21 *transcription start site and include the majority of the *p21 *coding regions in their final spliced products [35], and a long non-coding antisense RNA (*bx332409*) that regulates local epigenetic states [36] (Figure 2).

The *p21 *locus encodes two tiRNA clusters, one at the TSS (tss-tiRNAs) and the other at the CTCF-RNAPII site (CTCF proximal (cp)-tiRNAs) that are antisense to one another. The tss-tiRNAs are sense to the gene (as observed generally), while the cp-tiRNAs are antisense. Both overlap distinct peaks of RNAPII binding, suggesting that their biogenesis is tied to RNAPII molecules heading in opposite directions, possibly linked to nucleosome position [4], and this reinforces our previous finding that tiRNAs are found at sites of active RNAPII transcription initiation outside of canonical transcription start sites (Figure 2).

**Figure 3:**
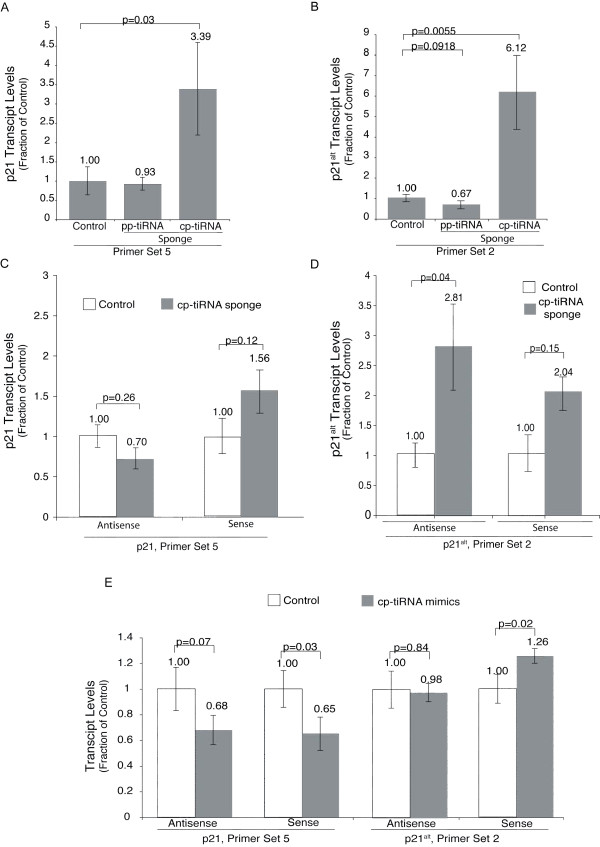
**The effects of transcription initiation (ti)RNA sponges and mimics on *p21 *(cyclin-dependent kinase inhibitor 1A gene, also known as *CDKN1A*) expression**. **(a) **Transfection by the CCCTC-binding factor (CTCF) proximal (cp)-tiRNA sponge resulted in an increase in *p21 *mRNA and **(b) ***p21^alt ^*transcript levels. Reverse transcription was non-specifically primed and cDNA was analyzed by quantitative (qPCR) using primer set 2 for *p21 *mRNA and primer set 1 for *p21^alt ^*RNA. **(c) **Further validation of the results in (a) and (b) were confirmed by transfection with the cp-tiRNA sponge followed by strand-specific quantitative reverse transcription (qRT)-PCR which showed an increase in both antisense and sense *p21^alt ^*transcript levels, and **(d) **an increase in sense *p21 *mRNA. **(e) **Transfection of cp-tiRNA mimics resulted in a decrease in both sense classical and alternate *p21 *transcripts. (a-e) Samples were analyzed at 72 h post transfection. The averages of samples transfected in triplicate are shown, error bars represent the standard errors of the means, and *P *values from paired t tests are shown.

To investigate the function of p21 tiRNAs, we utilized short antisense 'sponge' RNAs [37] that were designed to bind and inhibit tss-tiRNAs and cp-tiRNAs (Figure 2). MCF-7 cells transfected with the cp-tiRNA sponge demonstrated a significant increase of *p21 *mRNA and *p21^alt ^*expression, as measured by quantitative PCR (qPCR) (Figure 3a, b). In contrast, the tss-tiRNA sponge did not exhibit a detectable effect on *p21 *expression (Figure 3a, b), and thus cp-tiRNAs became the focus of the remainder of this study.

As reverse transcription in the qPCR samples was not specifically primed (Figure 3a, b), these transcripts might represent sense and/or antisense transcripts associated with these regions [36] or any of the plethora of splice variants. To determine the extent of the effect that the cp-tiRNA sponge has on relative sense and antisense *p21 *transcript levels, strand-specific reverse transcription PCR (RT-PCR) was performed. Upon treatment with the cp-tiRNA sponge, *p21 *mRNA, sense *p21^alt^*, and antisense *p21^alt ^*transcript levels increased, whereas transcripts antisense to *p21 *mRNA were unaffected (Figure 3c, d). These data indicate that CTCF-proximal tiRNAs may be involved in the negative regulation of p21.

We next performed the reciprocal experiment testing the effect that overexpression of CTCF-proximal tiRNA mimics has on p21 expression. Consistent with our speculation that tiRNAs are connected to transcriptional regulation, we found that overexpression of a set of four cp-tiRNA mimics resulted in a marked reduction of the *p21 *mRNA (Figure 3e). To confirm that the effect of the cp-tiRNA sponges and mimics was not restricted to MCF-7 cells we repeated these experiments in THP-1 cells and found that the principal results were recapitatulated (Additional file 6, Figure S4), indicating that cp-tiRNAs have a regulatory effect on *p21 *transcription in multiple human cell systems.

**Figure 4:**
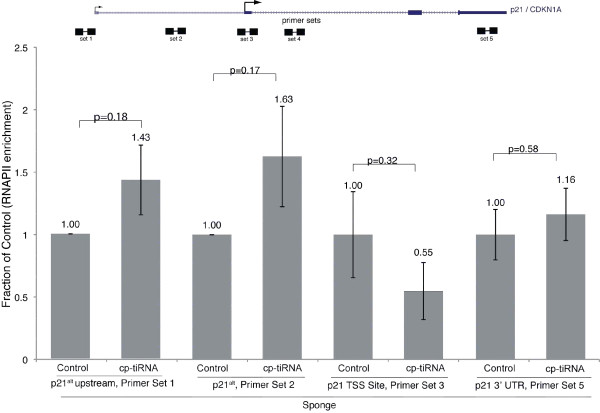
**RNA polymerase II (RNAPII) response to the *p21 *(cyclin-dependent kinase inhibitor 1A gene, also known as *CDKN1A*) CCCTC-binding factor (CTCF) proximal (cp)-transcription initiation (ti)RNA sponge**. Active RNAPII enrichment sites in the *p21 *(*CDKN1A*) locus were determined by chromatin immunoprecipitation (ChIP). RNAPII levels are generally unaffected, although a modest increase in RNAPII activity downstream of the *p21^alt ^*transcription start site was observed.

To further investigate the effects of cp-tiRNA sponge and mimics on p21 transcription, elongating forms of RNAPII were assessed by chromatin immunoprecipitation-PCR (ChIP-PCR). The only signal increase appeared in regions overlapping *p21^alt^*, although that increase was modest (Figure 4), suggesting that cp-tiRNAs do not function by affecting local RNAPII densities, but rather by directly or indirectly modulating local chromatin architecture.

**Figure 5:**
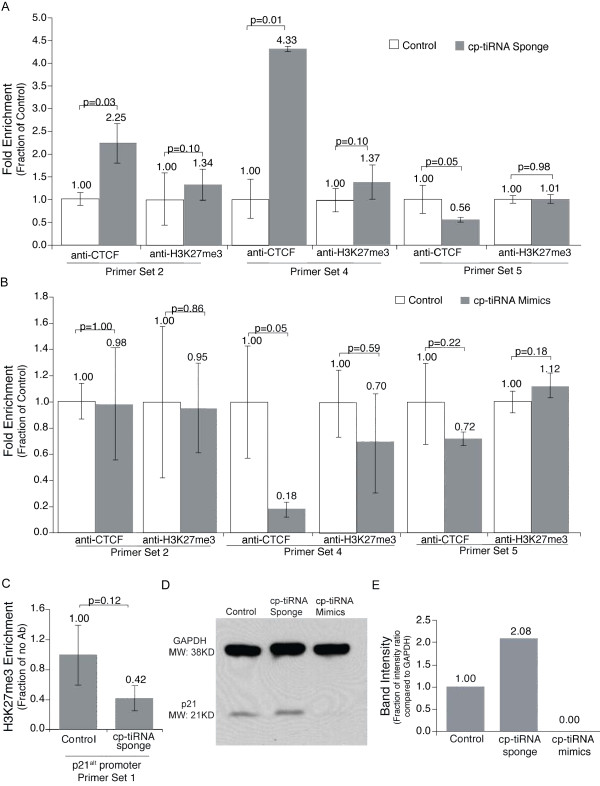
**CCCTC-binding factor (CTCF) binding and H3K27 trimethylation (H3K27me3) at *p21 *(cyclin-dependent kinase inhibitor 1A gene, also known as *CDKN1A*) in response to the CTCF proximal (cp)-transcription initiation (ti)RNA sponge and mimics**. **(a) **Transfection with the cp-tiRNA sponge resulted in an enrichment of CTCF at the conserved CTCF binding site. H3K27me3 was unaffected at the canonical *p21 *promoter or in the *p21*-coding region. **(b) **Transfection with cp-tiRNA mimics resulted in a decrease of CTCF at the CTCF binding site. H3K27me3 was generally unaffected. **(c) **Transfection with the cp-tiRNA sponge resulted in a decrease of H3K27me3 at the alternate p21 promoter, consistent with an increase in transcriptional activity. **(d) **As measured by western blot, samples transfected with the cp-tiRNA sponge showed an increase of p21 protein levels, while samples transfected with the cp-tiRNA mimics showed a decrease. **(e) **Differences in p21 protein levels from the western blot in (d) quantified using ImageJ. (a-d) Samples were analyzed as indicated 72 h post transfection. No antibody values were subtracted from each immunoprecipitation, and the resultant values were standardized to the input each sample. The averages of samples transfected in triplicate are shown, error bars represent the standard errors of the means, and *P *values from paired t tests are shown.

To explore this possibility we examined the effects of cp-tiRNAs sponge and mimic constructs on CTCF localization, and on epigenetic marks at various locations within the *p21 *locus by ChIP. The density of the silent state chromatin mark, H3K27me3, did not change upon introduction of cp-tiRNA sponge or mimic constructs at their perfectly complementary target sites (that is, at sites of tiRNA biogenesis; Figure 5a, b), as would be expected if the cp-tiRNA mimic or sponges were themselves altering local chromatin status, as has been observed previously with small non-coding RNAs associated with transcriptional gene silencing [38]. However, H3K27me3 levels upstream of the *p21^alt ^*transcription start site were decreased upon cp-tiRNA sponge treatment (Figure 5c). Given that the distance between the site of tiRNA biogenesis and the *p21^alt ^*promoter is greater than 6 kilobases, we speculated that these effects are facilitated by tiRNA-mediated regulation of other epigenetic regulators capable of acting at long distances.

Consistent with this, treatment with the cp-tiRNA sponge resulted in a significant increase in CTCF binding (Figure 5a), and overexpression of the cp-tiRNA mimics exhibited a significant decrease of CTCF binding (Figure 5b). This indicates that the effect of cp-tiRNAs on *p21 *transcription is directly related to its ability to modulate CTCF binding, which may be involved in three-dimensional (re)ordering of the *p21 *locus. Indeed, western blot analysis showed that p21 protein levels were increased in samples treated with the cp-tiRNA sponge, and decreased in samples treated with the cp-tiRNA mimic constructs (Figure 5d, e). Taken together, these data suggest that one function of *p21 *cp-tiRNAs may be to inhibit CTCF binding to the p21 gene, possibly as a means to repress transcription and downstream translation.

**Figure 6:**
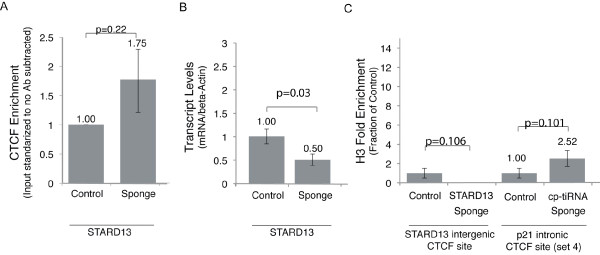
**The effect of a CCCTC-binding factor (CTCF) proximal (cp)-transcription initiation (ti)RNA sponge on StAR-related lipid transfer domain containing 13 (*STARD13*)**. **(a) **The effect of the *STARD13 *sponge on CTCF at the CTCF binding site. **(b) **The effects the *STARD13 *cp-tiRNA sponge on mRNA expression. Experiments were standardized to pCDNA transfected MCF-7 cells. **(c) **The effects of the *p21 *(cyclin-dependent kinase inhibitor 1A gene, also known as *CDKN1A*) cp-tiRNA and *STARD13 *tiRNA sponges on nucleosomal positioning based on histone H3 localization. (a-c) Samples were analyzed as indicated 72 h post transfection. The averages of triplicate transfected samples are shown with the error bars representative of the standard errors of the means, and *P *values from paired t tests. (a, c) No antibody values were subtracted from each IP, and the resultant values were standardized to the input for each sample.

**Figure 7:**
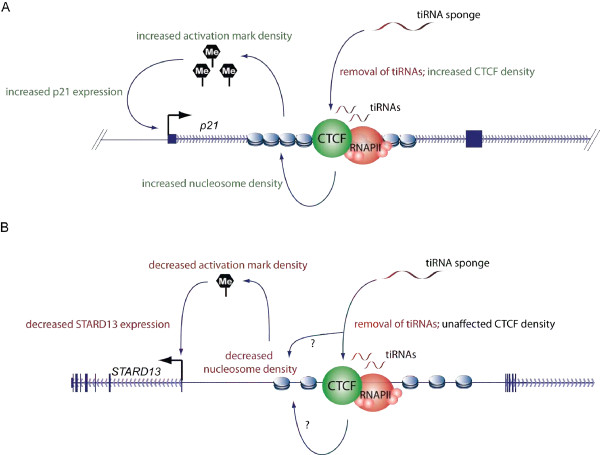
**A schematic representation of transcription initiation (ti)RNA sponge effects on the *p21 *(cyclin-dependent kinase inhibitor 1A gene, also known as *CDKN1A*) and StAR-related lipid transfer domain containing 13 (*STARD13*) loci**. **(a) **The p21 CCCTC-binding factor (CTCF) proximal (cp)-tiRNA sponge removes tiRNAs from the system, which in turn facilitates increased CTCF binding and, consistent with the literature, increased nucleosome ordering and density. CTCF-adjacent nucleosomes are known to be enriched for the H3K4 trimethylation (H3K4me3) transcription activation mark. Here we speculate that the increased density of this mark may drive the increased *p21 *expression observed in the presence of the cp-tiRNA sponge. **(b) **We observed little effect of the *STARD13 *sponge on CTCF density, but our results indicate that the sponge nonetheless induces reduced nucleosome density and *STARD13 *expression. Here we propose that reduced levels or concentrations of H3K4me3 marks due to the decrease in nucleosome density may modulate *STARD13 *expression. (a, b) Black, green and red text indicates no change, increased levels or decreased levels, respectively.

To test whether cp-tiRNAs can modulate CTCF binding at other loci we generated sponges for cp-tiRNAs derived from an intergenic region downstream of the *C2orf42 *(*Homo sapiens *chromosome 2 open reading frame 42), and an intergenic site upstream of StAR-related lipid transfer domain containing 13 (*STARD13*) (Additional file 7, Figure S5). To ensure that selection bias did not affect our study, these sites were chosen at random from approximately 900 sites with strong CTCF binding and tiRNA conservation across cell lines (see Methods). Examination of the *C2orf42 *site revealed no significant effect of tiRNA sponges (Additional file 8, Figure S6). However, we observed that *STARD13 *cp-tiRNA sponges resulted in a reduction in *STARD13 *mRNA expression, in spite of the fact that CTCF binding was largely unaffected (Figure 6a, b). This cp-tiRNA-mediated sponge effect is contrary to that observed for p21, which strongly increased p21 expression. To further investigate this we examined local nucleosome density at both loci and found that the *p21 *cp-tiRNA sponges induced increased nucleosomal localization, while the *STARD13 *cp-tiRNA induced a decrease in nucleosomal localization (Figure 6c). This is consistent with our hypothesis that cp-tiRNAs mimics and sponges facilitate condition dependent small-scale rearrangements to nucleosome order, and that this in turn leads to large-scale chromatin reorganization orchestrated by CTCF or other DNA binding and chromatin modifying complexes. Indeed, recent work has shown that an array of up to 20 well positioned nucleosomes enriched for the transcription initiation mark H3K4me3 flank CTCF sites, a phenomenon previously only observed downstream of TSSs [39]. This finding not only potentially explains why tiRNAs are frequently found at CTCF sites, but also suggests that the contradictory *p21 *and *STARD13 *tiRNA sponge effects may result from changes to the local density of chromatin activating marks (Figure 7).

The mechanism by which tiRNAs inhibit CTCF localization is unclear, although there are several obvious possibilities: (i) cp-tiRNAs spanning the CTCF binding site may coat local chromatin by binding nascent transcripts [36] or chromatin associated RNAs [40-42], which could sterically hinder CTCF from accessing its binding site; (ii) cp-tiRNAs may directly interact with CTCF and inhibit CTCF binding, although attempts to immunoprecipitate CTCF with biotin-linked cp-tiRNAs were unsuccessful (data not shown); (iii) cp-tiRNAs may bind to regulatory elements including cis-acting ncRNAs (for example, *bx332409 *at *p21*) or polycomb group components and direct their action to specific sites; or (iv) cp-tiRNAs may serve as sequence-specific markers for chromatin modification complexes.

## Conclusions

The data presented here indicate that cp-tiRNAs can have a powerful effect on CTCF binding and local transcription. Indeed, tiRNA-mediated modulation of CTCF binding at *p21 *not only reduces *p21 *mRNA and protein levels, but also appears to affect chromatin state and expression of *p21^alt ^*transcripts. This suggests that at some loci the role of tiRNAs, whose biogenesis is connected to RNAPII activity and progression, may be to modulate (presumably indirectly) local chromatin states, which in turn regulates the binding of other factors including CTCF. Indeed, the relationship between tiRNAs and epigenetic structures may indicate a self-reinforcing feedback loop wherein the RNAPII-nucleosome interaction generates tiRNAs, which in turn serve to mark (directly or indirectly) nucleosome positions and/or epigenetic state. Although the mechanism of tiRNA action is still elusive, this work is the first to report a role for tiRNAs in gene regulation, and shows that at least a subset of tiRNAs are functional modulators of CTCF, which may lead to the development of novel RNA-based therapeutics that target epigenetic regulation of gene structure and transcription.

## Methods

### Bioinformatic analyses

Bioinformatic analyses were performed on a local high-performance computer at the UQ Institute for Molecular Bioscience that houses a mirror of the UCSC Genome Brower [43]. We used a suite of in-house AWK, C, Perl, and Python scripts and UCSC backend tools. All small RNA, CTCF binding and RNAPII binding data were obtained from publicly available sources and are listed in detail in Additional file 1, Table S1. For all ChIP-seq datasets the available peak calls were used, except in the case of the mES RNAPII data where peaks were defined as regions with signal greater than 3 SDs from the mean. All intersections were performed using a modified version of the UCSC backend tools bedIntersect or overlapSelect. A minimum of 1 bp of overlap was required, but generally >50% of any given feature intersected with another. The relative enrichment of small RNAs at CTCF sites with or without RNAPII coverage was computing using an in-house (Perl) bootstrapping program over 1,000 iterations as previously described [9]. Bootstrapping was constrained such that the randomized placements of small RNAs excluded TSSs, known small RNA annotations, repeat masker annotations and genome assembly gaps. For the analysis of the MCF-7, mES, and human CTCF data grouped into 'cancer' and 'normal' the data was parsed so that data points that intersected with known small RNA annotations, the 500 bp adjacent to TSSs, repeat masker annotations or Ensembl annotations less than 300 nucleotides were removed. The conserved CTCF site data set were generated by taking all the peaks identified by Broad/ENCODE as significant across all eight sources (see Additional file 1, Table S1) and intersecting them against one another using the UCSC backend tool overlapSelect. Overlapping CTCF features (called peaks) were collapsed into one concordant set of coordinates using the UCSC tool featureBits. Small RNA size distributions were computed as previously described [9].

### Cell culture and transfection

Experiments were conducted in MCF-7 and THP-1 cells cultured at 37°C and 5% CO_2 _in Dulbecco's modified Eagle's medium or RPMI 1640 (THP-1 cells only) supplemented with 10% fetal bovine serum and penicillin-streptomycin (Life Technologies, Carlsbad, California, USA). Plasmid transfections of MCF-7 cells were performed using Lipofectamine 2000 (Life Technologies, Carlsbad, California, USA) per the manufacturer's guidelines at a concentration of 1 μg/1 × 10^6 ^cells. Plasmid transfections of THP-1 cells were carried out using the Neon electroporation system (Life Technologies, Carlsbad, CA, USA), per manufacturer's guidelines.

### Sponge and mimic plasmid construction

Sponge plasmids: reverse complement sequences to the promoter-proximal and CTCF-proximal tiRNA sites in p21 were amplified from MCF-7 genomic DNA by polymerase chain reaction (PCR) using primers with *Bgl*II restriction sites on either end (Additional file 3, Table S2). Amplified DNA was digested by *Bgl*II and ligated into the similarly cut pCDNA3 U6M2 plasmid. The parent vector, pCDNA3 U6M2, was used as a control in sponge experiments. The resulting sponge sequences were: p21 promoter-proximal tiRNA sponge (pp-tiRNA) 5'CCGCCGGCCCGGGGTCCCCTGTTGTCTGCCGCCGCTCTCTCACCTCCTCTGAGTGCCTCGGT GCCTCGGCGAATCCGCGCCCAGCTCCGGCTCCACAAGGAACTGACTTCGGCAGCTGCTCACA CCTCAGCTGG3' and p21 CTCF-proximal tiRNA sponge (cp-tiRNA) 5'GGGCTCAGGGCTTCCCGACCTAGCCCAGATTCCCTCTCCGAAAGCTACAGGGCTGAGCGGA GCAGGGGGGCGAGTCGCCCCCTGGGGCGCCGCCGCCTGGCGCGGACCACAGCGCGTCCTCT CCGTCCCAAACCCCTGGGGGACACTTGCGCCCTCTTCGTGAGGAAAAGCATCTTGGAGCTGG GTTAGGAACTTGGGGCGCCCAGGCAGCTTCCCCTCTCCTTGCCTCCCTCCACGTCGCGTTTCT GGGAGGACTTGCGAGCGGTTTTGTTTTCGTTGCTCCCGTCTATTTTTATTTTCCAGGGATCTGA CT3'.

Sponge constructs for C2Orf41 and STARD13 were constructed by annealing the respective oligonucleotides together (Additional file 3, Table S2), top strand oligo (200 μM): 5 μl bottom strand oligo (200 μM): 5 μl, 10 × Oligo Annealing Buffer (BLOCK-iT kit*, Life Technologies, Carlsbad, California, USA): 2 μl, DNase/RNase-free water: 8 μl. The reaction was heated to 95°C for 4 min, cooled to room temperature and treated to polynucleotide kinase (PNK) (1 μl PNK (10,000 U/ml), 1 μl T4 ligase buffer, 4 μl dsOligo, 4 μl water, at 37°C for 30 min). The oligonucleotides were purified using minelute PCR purification kit and ligated to pCDNA3 U6M2 as described above.

### Mimic plasmids

A negative control and four sequences of CTCF-proximal tiRNAs were synthesized (Integrated DNA Technologies) and cloned into the BLOCK-iT U6 RNAi Entry Vector (Life Technologies, Carlsbad, CA, USA) per manufacturer's guidelines. The mimics, four predominant cp-tiRNA sequences (Additional file 9, Table S3) were independently cloned into the U6 driven BLOCK-iT system (Life Technologies, Carlsbad, CA, USA). The resultant plasmids were transfected into MCF-7 cells as described previously [36].

### Quantitative strand-specific PCR (qPCR)

RNA was extracted (RNeasy Qiacube, Qiagen), DNase treated (TURBO DNase, Ambion), reverse transcribed (Reverse Transcriptase Core Kit, Eurogentec) using the non-specific or indicated primers (for strand-specific RT-PCR), and analyzed by qPCR using indicated primers (Kapa Sybr Fast Universal qPCR Kit, Kapa Biosystems, Woburn, MA, USA) (Additional file 9, Table S3). In strand-specific RT-PCR, reverse transcription is primed with a gene specific forward or reverse primer alone, thereby generating cDNA of specifically the antisense or sense strand of the targeted region respectively. Controls for this assay are reverse transcription or template RNA in the absence of any primer. Quantitative PCR (qPCR) is then performed using forward and reverse primers, yielding amplicons that represent sense or antisense transcripts overlapping that region with the control no primer RT sample values subtracted as background from the directional RT primed samples.

### ChIP

ChIP assays were performed as previously described [44]. DNA was immunoprecipitated using anti-RNAPII phosphor-S2 (AbCam no. ab5095, AbCam Cambridge, MA USA), anti-H3K27me3 (Cell Signaling no. 9756S, Danvers, MA, USA), or anti-CTCF (Santa Cruz Biotechnology no. sc-15914, Santa Cruz, CA, USA) antibody bound complexes were then pulled down using magnetic Dynabeads Protein A (Life Technologies, Carlsbad, CA, USA). DNA was then recovered by phenol/chloroform extraction and analyzed by qPCR using indicated primers (Additional file 9, Table S3) (Kapa Sybr Fast Universal qPCR Kit, Kapa Biosystems, Woburn, MA, USA).

### Western blot

Cells were lysed in modified RIPA buffer (25 mM Tris HCl, pH 7.5, 15 mM NaCl, 1% Nonidet P-40, 1% NaD, and 0.1% SDS) and separated on a NuPAGE 4% to 12% BisTris gel (Life Technologies, Carlsbad, CA, USA). Proteins were transferred to a nitrocellulose membrane which was blocked with 5% milk for 1 h and then incubated overnight at 4°C with anti-p21 (Cell Signaling no. 2946) and anti-glyceraldehyde 3-phosphate dehydrogenase (GAPDH) (Millipore no. MAB374, Billerica, MA, USA) antibodies. The membrane was then washed (10 mM Tris HCl, pH 7.5, 50 mM NaCl, 0.075% Tween 20) and incubated with an anti-mouse horseradish peroxidase-conjugated secondary antibody for 1 h at room temperature (Upstate no. 12-349, Billerica, MA, USA). The membrane was then washed, treated with chemiluminescent detection reagent (HyGLO, Denville Metuchen, NJ, USA), and exposed to film. Blot density of a binary image of Figure 5d was calculated using ImageJ. Results were standardized to GAPDH and expressed as fractions of control values.

## Competing interests

RJT and JSM have an ownership stake in a patent concerning tiRNAs and their diagnostic and therapeutic uses (International Patent No. AU2009/000423).

## Authors' contributions

RJT performed the bioinformatic analyses, helped design the laboratory experiments and wrote the manuscript. PGH performed the generated and tested tiRNA mimics and sponges and helped write the manuscript. JSM assisted in study design and direction. KVM designed the laboratory experiments, performed tiRNA sponge and mimic experiments and wrote the manuscript. All authors read and approved the final manuscript.

## Supplementary Material

Additional file 1 **Table S1**. Small RNA (sRNA), CCCTC-binding factor (CTCF) chromatin immunoprecipitation (ChIP)-seq and RNA polymerase II (RNAPII) ChIP-seq datasets. ^‡^Data obtained from ENCODE/UCSC and can be retrieved from the publicly available ENCODE UCSC genome browser (http://genome.ucsc.edu/ENCODE/) [5,9]. *All data sets with identifiers beginning with GSE can be found at that the NCBI Gene Expression Omnibus (http://www.ncbi.nlm.nih.gov/geo/). MCF-7 CTCF ChIP-seq data can be found in the EBI Array Express Archive (http://www.ebi.ac.uk/arrayexpress/). Click here for file

Additional file 2 **Figure S1**. Enrichment of transcription initiation (ti)RNAs at sites of conserved CCCTC-binding factor (CTCF) binding genome-wide. CTCF sites were obtained from the ENCODE Broad Institute UCSC Histone Mods tracks. Only those with significant peaks conserved across GM12878, HepG2, HMEC, HSMM, HUVEC, K562, NHEK and NHLF cell lines were considered in this analysis. **(a) **Enrichment of small RNAs at all conserved CTCF sites (gray), and conserved CTCF sites with evidence of conserved RNA polymerase II (RNAPII) binding in HUVEC, K562 and NHEK cells (black). **(b-d) **The size distribution of small RNAs found at conserved CTCF sites with evidence of RNAPII binding in (b) THP-1, (c) 5-8f and (d) MCF cells. In (b) and (c) nuclear small RNAs are shown in black, and cytoplasmic small RNAs are depicted in gray. Click here for file

Additional file 3 **Table S2**. Conserved CCCTC-binding factor (CTCF) and CTCF-RNA polymerase II (RNAPII) site intersection with small RNAs (sRNAs). Click here for file

Additional file 4 **Figure S2**. Enrichment of transcription initiation (ti)RNAs at sites of subsets of conserved CCCTC-binding factor (CTCF) binding sites genome-wide. CTCF sites were parsed to (i) exclude any that mapped within 500 bp of a TSSs or overlapped repeat masker, small RNA or Ensembl gene annotations less than 300 nucleotides, (ii) into groups by cell type where 'cancer' was derived from MCF-7, K562 and HepG2 data and normal was derived from GM12878, HUVEC, HMEC, HSMM, NHEK and NHLF data, and (iii) intersected with the most robust RNA polymerase II (RNAPII) data for each group (MCF-7 and HUVEC, for cancer and normal, respectively). Note that tiRNAs are highly enriched at CTCF-RNAPII sites even in these highly reduced sets. Click here for file

Additional file 5 **Figure S3**. The intronic *p21 *(cyclin-dependent kinase inhibitor 1A gene, also known as *CDKN1A*) CCCTC-binding factor-RNA polymerase II (CTCF-RNAPII) site is highly conserved in mammals. In the top panel the *p21/CDKN1A *locus in mouse is shown. The bottom panel is a focused view of the intronic CTCF-RNAPII site. Note the conserved CTCF site, RNAPII binding, and high conservation of the site itself and the sequence immediately to the left (5' with respect to *p21*), which is the site of transcription initiation (ti)RNA biogenesis in humans. Click here for file

Additional file 6 **Figure S4**. *p21 *(cyclin-dependent kinase inhibitor 1A gene, also known as *CDKN1A*) CCCTC-binding factor (CTCF) proximal (cp)-transcription initiation (ti)RNA RNA sponge and mimics effects are conserved in THP-1 cells. Samples were prepared and analyzed identical to those shown in Figure 3. Note the increase in *p21 *mRNA levels in response to the cp-tiRNA sponge, and the decrease in expression in response to cp-tiRNA mimics. Click here for file

Additional file 7 **Figure S5**. A schematic of the CCCTC-binding factor (CTCF) sites proximal to *C2orf42 *and StAR-related lipid transfer domain containing 13 (*STARD13*). **(a, b) **Gene models are shown at the top of each panel, followed by the collapsed density of small RNAs in three datasets, CTCF binding density in nine human cell types, and RNA polymerase II (RNAPII) binding in three human cell lines. In (a) the conserved C2orf42 CTCF site is approximately 5 kb downstream of the 3' untranslated region (UTR). The CTCF site in (b) sits approximately halfway between *STARD13 *and *RFC3*. Click here for file

Additional file 8 **Figure S6**. The effect of the CCCTC-binding factor (CTCF) proximal (cp)-transcription initiation (ti)RNA RNA sponge on CTCF sites proximal to *C2orf42*. **(a) **The effects of the C2Orf42 sponge on CTCF localization. **(b) **The effects of *C2Orf41 *tiRNA sponges on mRNA expression. Experiments were standardized to pCDNA transfected MCF-7 cells. **(c) **The effects of the *C2Orf41 *sponge on histone H3 localization. Samples were analyzed as indicated 72 h post transfection. The averages of triplicate transfected samples are shown with the error bars representative of the standard errors of the means, and *P *values from paired t tests. (a, c) No antibody values were subtracted from each IP, and the resultant values were standardized to the input for each sample. Click here for file

Additional file 9 **Table S3**. Oligonucleotides used for cloning, quantitative reverse transcription (qRT)-PCR, and chromatin immunoprecipitation (ChIP) analysis. Click here for file
