# Supplementary material for: The relationship between transcription initiation RNAs and CCCTC-binding factor (CTCF) localization
Source: Epigenetics Chromatin. 2011 Aug 3;4:13. doi: 10.1186/1756-8935-4-13 (PMC3170176; doi:10.1186/1756-8935-4-13)
Supplement: Additional file 1 — Table S1. Small RNA (sRNA), CCCTC-binding factor (CTCF) chromatin immunoprecipitation (ChIP)-seq and RNA polymerase II (RNAPII) ChIP-seq datasets. ‡Data obtained from ENCODE/UCSC and can be retrieved from the publicly available ENCODE UCSC genome browser (http://genome.ucsc.edu/ENCODE/) [5,9]. *All data sets with identifiers beginning with GSE can be found at that the NCBI Gene Expression Omnibus (http://www.ncbi.nlm.nih.gov/geo/). MCF-7 CTCF ChIP-seq data can be found in the EBI Array Express Archive (http://www.ebi.ac.uk/arrayexpress/). [file 1756-8935-4-13-S1.PDF]

Cell type/line	sRNAs	CTCF	RNAPII	Notes & Sources
Human cancer				
THP-1	x			*Monocytic leukemia cell line*<br>GSE20664 [1]
5-8f	x			*Nasopharyngeal carcinoma cell line*<br>GSE22918 [2]
MCF-7	x	x	x	*Breast cancer cell line*<br>sRNAs: http://www.illumina.com/landing/idea/<br>(Illumina Research and Development)<br>CTCF: EBI E-TABM-828 [3]<br>RNAPII: GSE23701 [4]
K562‡		x	x	*Myelogenous leukemia cell line*<br>CTCF: wgEncodeBroadChipSeqPeaksK562Ctcf<br>RNAPII: wgEncodeBroadChipSeqPeaksK562Pol2b
HepG2‡		x		*Liver hepatocellular carcinoma*<br>CTCF: wgEncodeBroadChipSeqPeaksHepg2Ctcf
Normal human tissues				
GM12878‡		x	x	*Lymphoblastoid cell line*<br>RNAPII: GSE19550 [5]<br>CTCF: wgEncodeBroadChipSeqPeaksGM1278Ctcf
HUVEC‡		x	x	*Human umbilical vein endothelial cells*<br>CTCF: wgEncodeBroadChipSeqPeaksHuvecCtcf<br>RNAPII: wgEncodeBroadChipSeqPeaksHuvecPol2b
HMEC‡		x		*Human mammary epithelial cells*<br>CTCF: wgEncodeBroadChipSeqPeaksHmecCtcf
HSMM‡		x		*Human skeletal muscle myoblast*<br>CTCF: wgEncodeBroadChipSeqPeaksHsmmCtcf
NHEK‡		x		*Normal human epidermal keratinocytes*<br>CTCF: wgEncodeBroadChipSeqPeaksNhekCtcf
NHLF‡		x		*Normal human lung fibroblasts*<br>CTCF: wgEncodeBroadChipSeqPeaksNhlkCtcf
Mouse embryonic stem cells				
mES	x	x	x	Mouse embryonic stem cells<br>sRNAs: GSE19894 [6]<br>CTCF: GSE27944 [7]<br>RNAPII: GSE16893 [8]

Table S1 References

1. Taft RJ, Simons C, Nahkuri S, Oey H, Korbie DJ, Mercer TR, Holst J, Ritchie W, Wong JJ, Rasko JE *et al*: **Nuclear-localized tiny RNAs are associated with transcription initiation and splice sites in metazoans**. *Nat Struct Mol Biol* 2010, **17**(8):1030-1034.

2. Liao JY, Ma LM, Guo YH, Zhang YC, Zhou H, Shao P, Chen YQ, Qu LH: **Deep sequencing of human nuclear and cytoplasmic small RNAs reveals an unexpectedly complex subcellular distribution of miRNAs and tRNA 3' trailers**. *PLoS One* 2010, **5**(5):e10563.

3. Schmidt D, Schwalie PC, Ross-Innes CS, Hurtado A, Brown GD, Carroll JS, Flicek P, Odom DT: **A CTCF-independent role for cohesin in tissue-specific transcription**. *Genome Res* 2010, **20**(5):578-588.

4. Joseph R, Orlov YL, Huss M, Sun W, Kong SL, Ukil L, Pan YF, Li G, Lim M, Thomsen JS *et al*: **Integrative model of genomic factors for determining binding site selection by estrogen receptor-alpha**. *Mol Syst Biol* 2010, **6**:456.

5. Myers RM, Stamatoyannopoulos J, Snyder M, Dunham I, Hardison RC, Bernstein BE, Gingeras TR, Kent WJ, Birney E, Wold B *et al*: **A user's guide to the encyclopedia of DNA elements (ENCODE)**. *PLoS Biol* 2011, **9**(4):e1001046.

6. Babiarz JE, Ruby JG, Wang Y, Bartel DP, Blelloch R: **Mouse ES cells express endogenous shRNAs, siRNAs, and other Microprocessor-independent, Dicer-dependent small RNAs**. *Genes Dev* 2008, **22**(20):2773-2785.

7. Martin D, Pantoja C, Fernandez Minan A, Valdes-Quezada C, Molto E, Matesanz F, Bogdanovic O, de la Calle-Mustienes E, Dominguez O, Taher L *et al*: **Genome-wide CTCF distribution in vertebrates defines equivalent sites that aid the identification of disease-associated genes**. *Nat Struct Mol Biol* 2011, **18**(6):708-714.

8. Goldberg AD, Banaszynski LA, Noh KM, Lewis PW, Elsaesser SJ, Stadler S, Dewell S, Law M, Guo X, Li X *et al*: **Distinct factors control histone variant H3.3 localization at specific genomic regions**. *Cell* 2010, **140**(5):678-691.

9. Rosenbloom KR, Dreszer TR, Pheasant M, Barber GP, Meyer LR, Pohl A, Raney BJ, Wang T, Hinrichs AS, Zweig AS *et al*: **ENCODE whole-genome data in the UCSC Genome Browser**. *Nucleic Acids Res* 2010, **38**(Database issue):D620-625.
