# Supplementary figures and images for: The relationship between transcription initiation RNAs and CCCTC-binding factor (CTCF) localization
Source: Epigenetics Chromatin. 2011 Aug 3;4:13. doi: 10.1186/1756-8935-4-13 (PMC3170176; doi:10.1186/1756-8935-4-13)

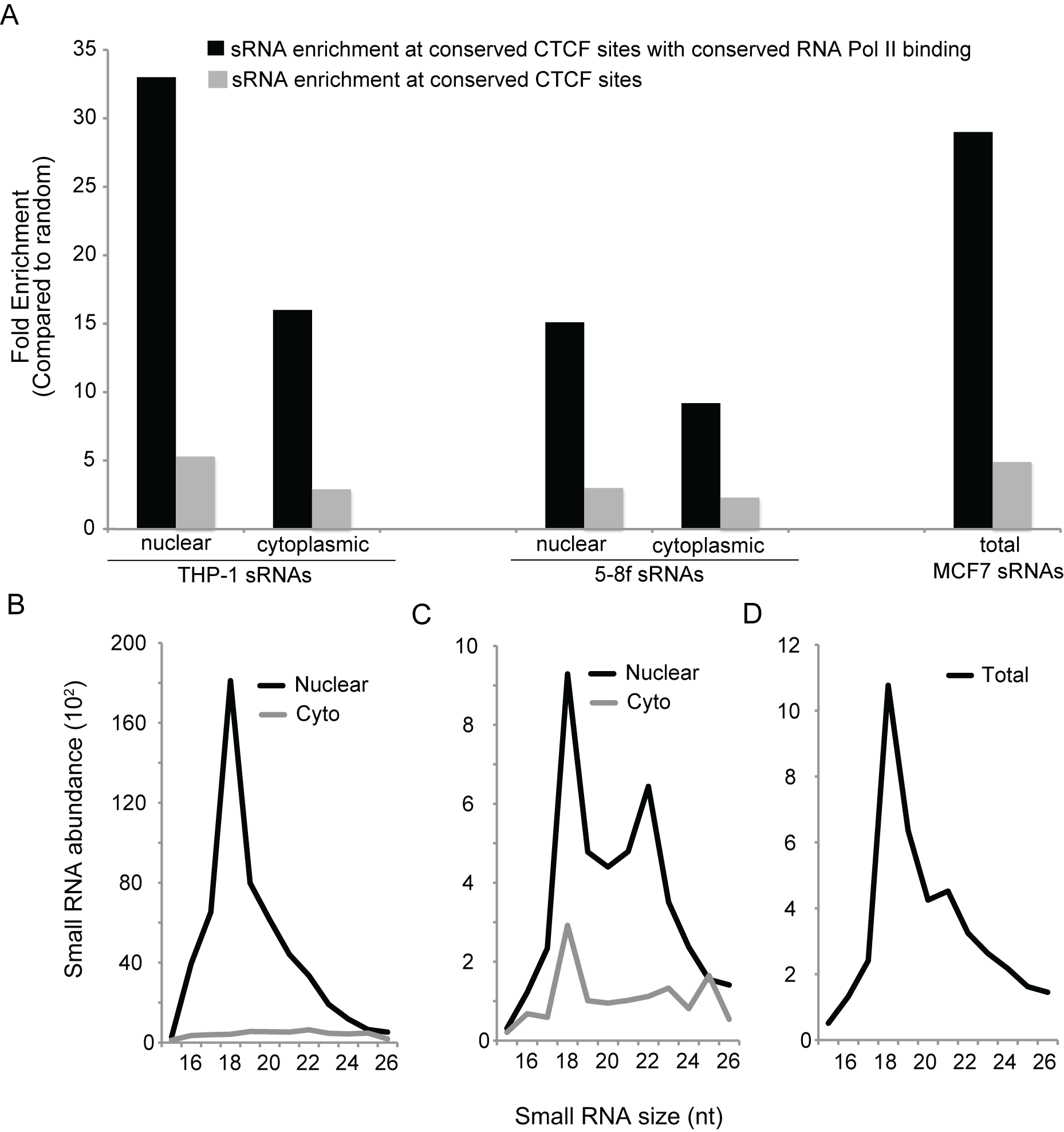

Supplement: Additional file 2 — Figure S1. Enrichment of transcription initiation (ti)RNAs at sites of conserved CCCTC-binding factor (CTCF) binding genome-wide. CTCF sites were obtained from the ENCODE Broad Institute UCSC Histone Mods tracks. Only those with significant peaks conserved across GM12878, HepG2, HMEC, HSMM, HUVEC, K562, NHEK and NHLF cell lines were considered in this analysis. (a) Enrichment of small RNAs at all conserved CTCF sites (gray), and conserved CTCF sites with evidence of conserved RNA polymerase II (RNAPII) binding in HUVEC, K562 and NHEK cells (black). (b-d) The size distribution of small RNAs found at conserved CTCF sites with evidence of RNAPII binding in (b) THP-1, (c) 5-8f and (d) MCF cells. In (b) and (c) nuclear small RNAs are shown in black, and cytoplasmic small RNAs are depicted in gray. [file 1756-8935-4-13-S2.PNG]

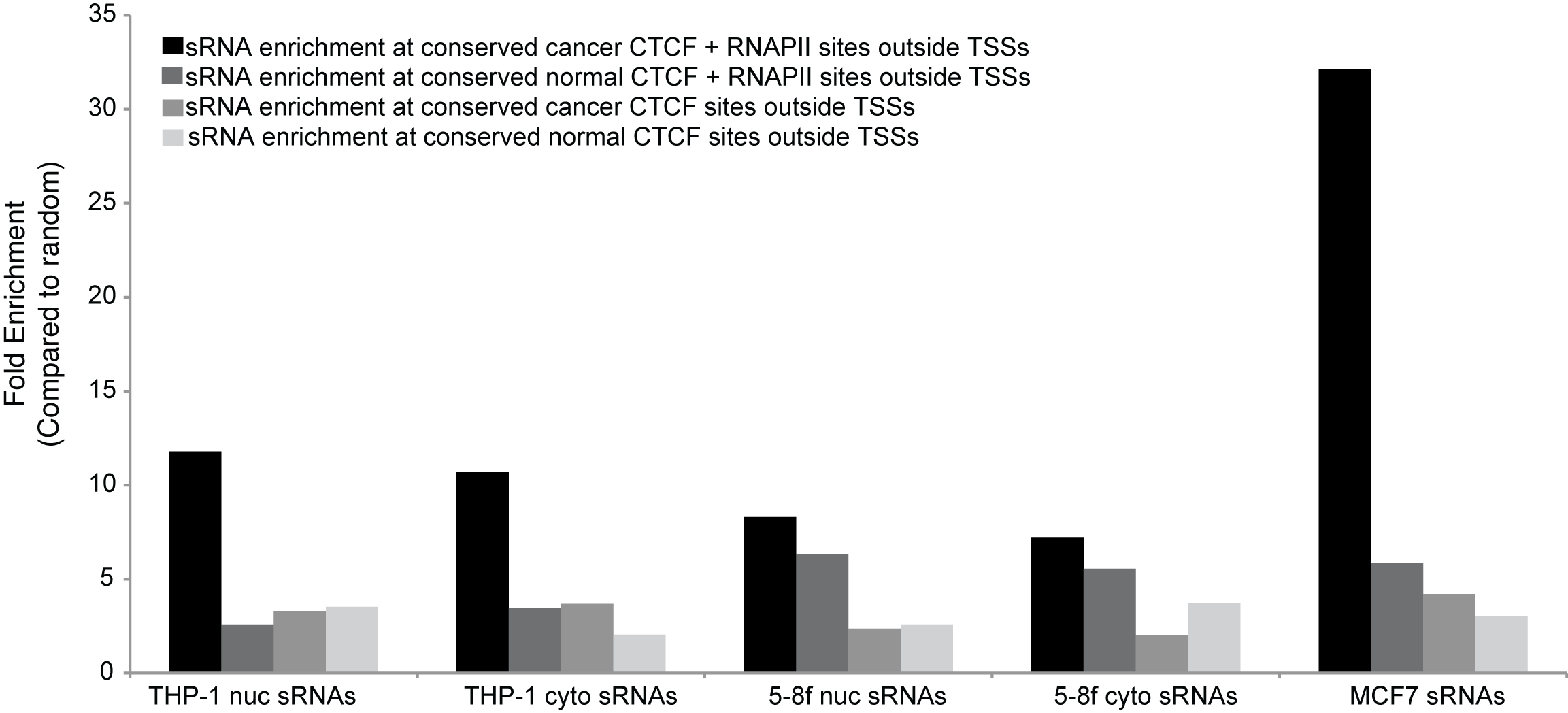

Supplement: Additional file 4 — Figure S2. Enrichment of transcription initiation (ti)RNAs at sites of subsets of conserved CCCTC-binding factor (CTCF) binding sites genome-wide. CTCF sites were parsed to (i) exclude any that mapped within 500 bp of a TSSs or overlapped repeat masker, small RNA or Ensembl gene annotations less than 300 nucleotides, (ii) into groups by cell type where 'cancer' was derived from MCF-7, K562 and HepG2 data and normal was derived from GM12878, HUVEC, HMEC, HSMM, NHEK and NHLF data, and (iii) intersected with the most robust RNA polymerase II (RNAPII) data for each group (MCF-7 and HUVEC, for cancer and normal, respectively). Note that tiRNAs are highly enriched at CTCF-RNAPII sites even in these highly reduced sets. [file 1756-8935-4-13-S4.PNG]

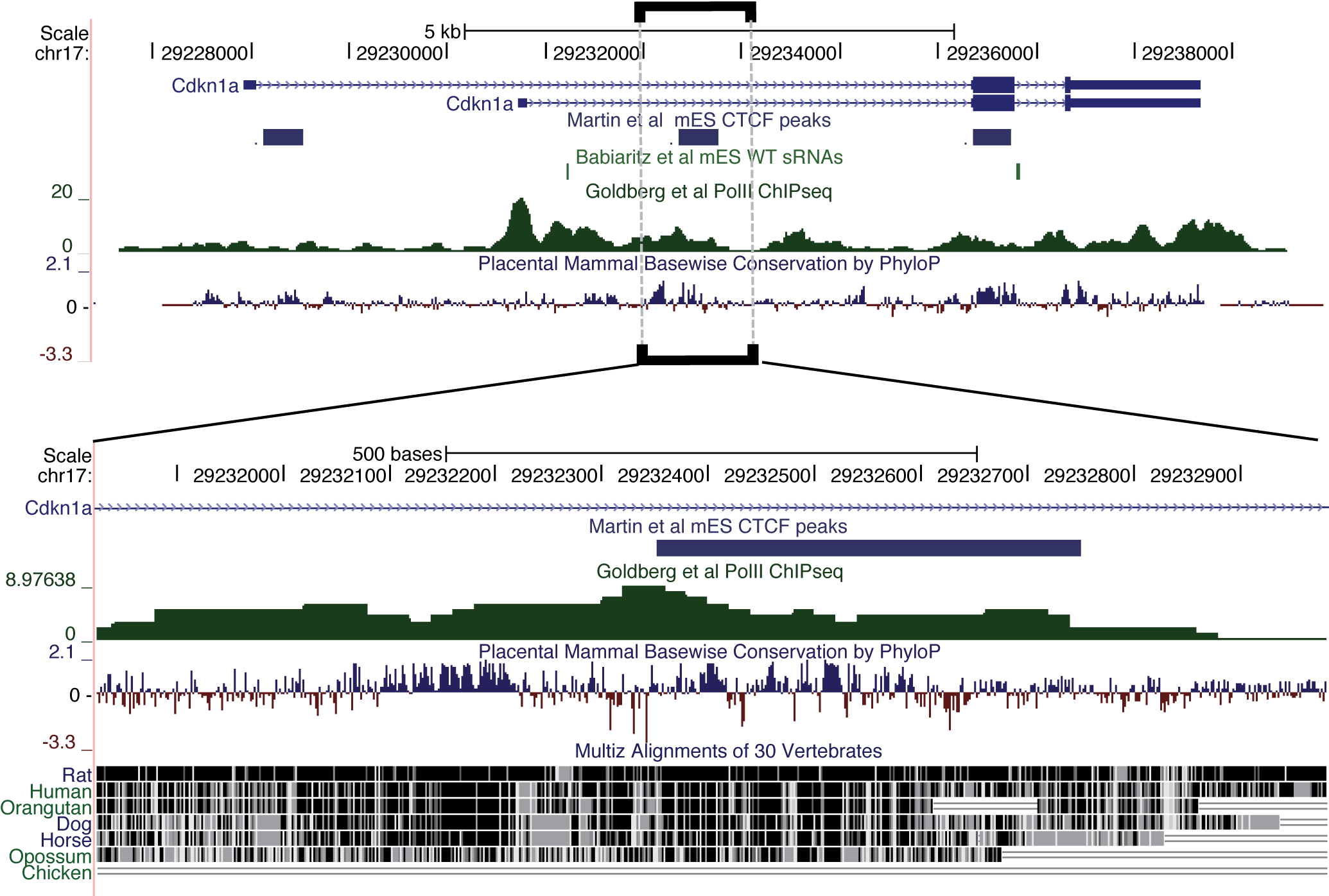

Supplement: Additional file 5 — Figure S3. The intronic p21 (cyclin-dependent kinase inhibitor 1A gene, also known as CDKN1A) CCCTC-binding factor-RNA polymerase II (CTCF-RNAPII) site is highly conserved in mammals. In the top panel the p21/CDKN1A locus in mouse is shown. The bottom panel is a focused view of the intronic CTCF-RNAPII site. Note the conserved CTCF site, RNAPII binding, and high conservation of the site itself and the sequence immediately to the left (5' with respect to p21), which is the site of transcription initiation (ti)RNA biogenesis in humans. [file 1756-8935-4-13-S5.PNG]

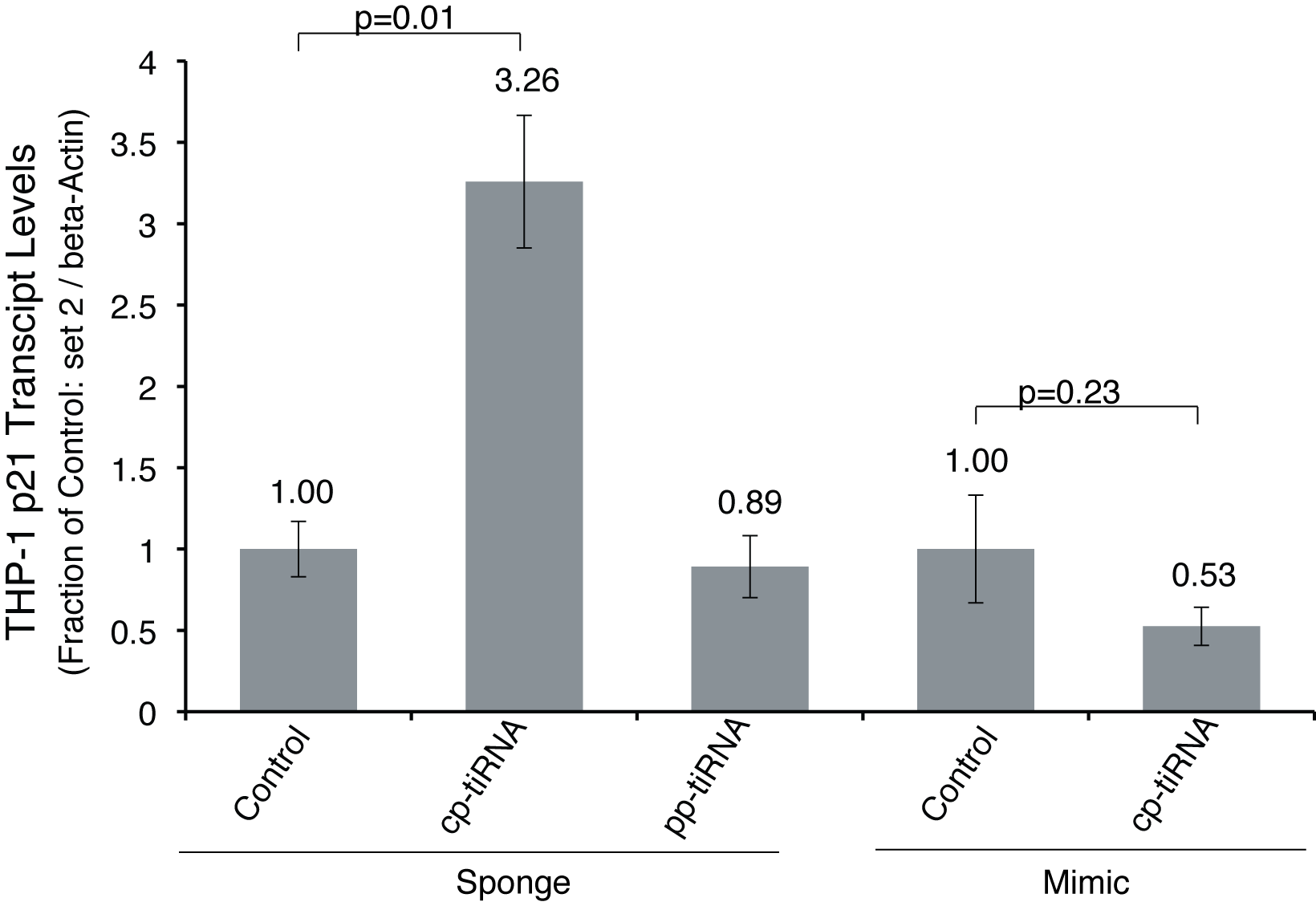

Supplement: Additional file 6 — Figure S4. p21 (cyclin-dependent kinase inhibitor 1A gene, also known as CDKN1A) CCCTC-binding factor (CTCF) proximal (cp)-transcription initiation (ti)RNA RNA sponge and mimics effects are conserved in THP-1 cells. Samples were prepared and analyzed identical to those shown in Figure 3. Note the increase in p21 mRNA levels in response to the cp-tiRNA sponge, and the decrease in expression in response to cp-tiRNA mimics. [file 1756-8935-4-13-S6.PNG]

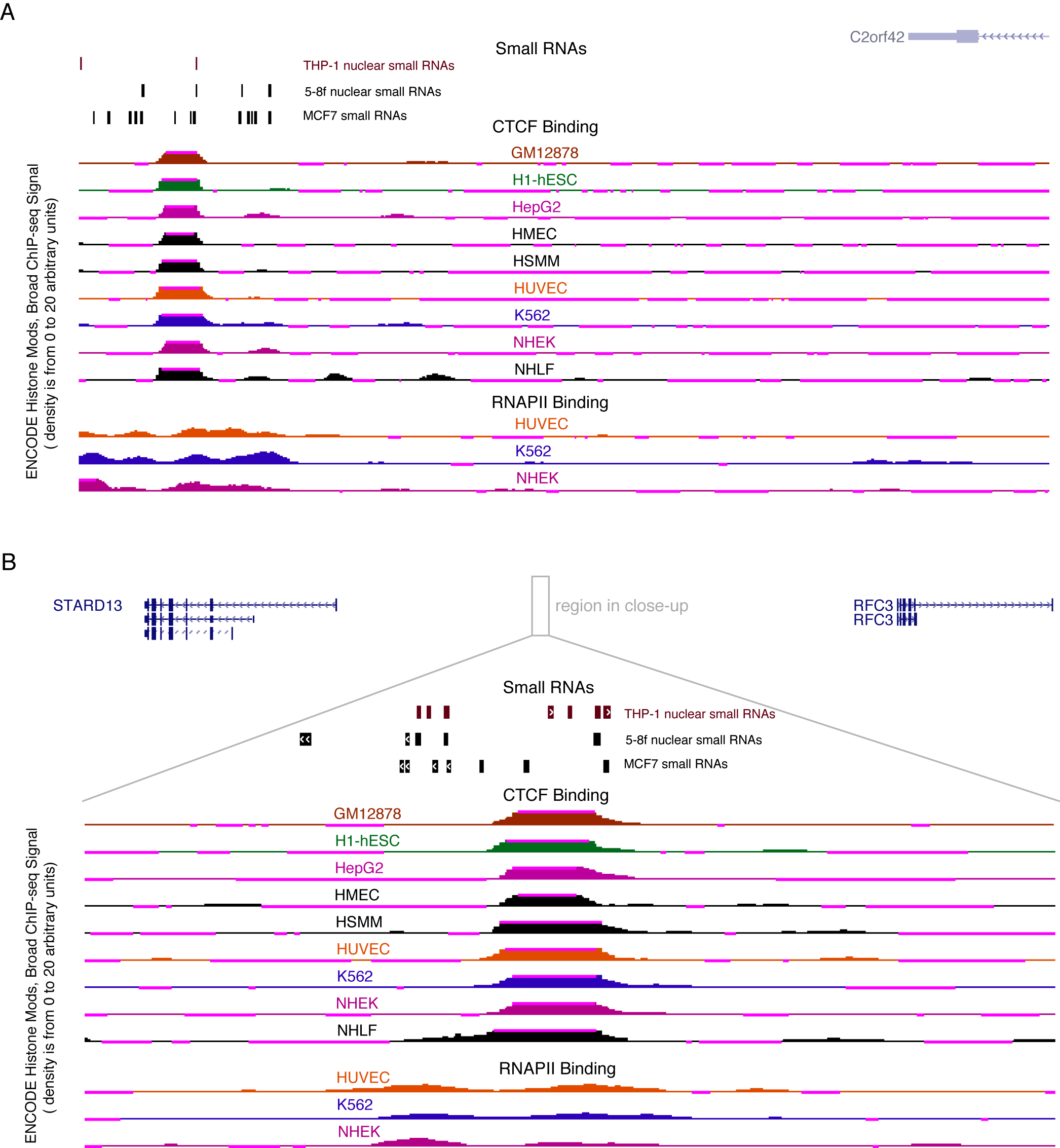

Supplement: Additional file 7 — Figure S5. A schematic of the CCCTC-binding factor (CTCF) sites proximal to C2orf42 and StAR-related lipid transfer domain containing 13 (STARD13). (a, b) Gene models are shown at the top of each panel, followed by the collapsed density of small RNAs in three datasets, CTCF binding density in nine human cell types, and RNA polymerase II (RNAPII) binding in three human cell lines. In (a) the conserved C2orf42 CTCF site is approximately 5 kb downstream of the 3' untranslated region (UTR). The CTCF site in (b) sits approximately halfway between STARD13 and RFC3. [file 1756-8935-4-13-S7.PNG]

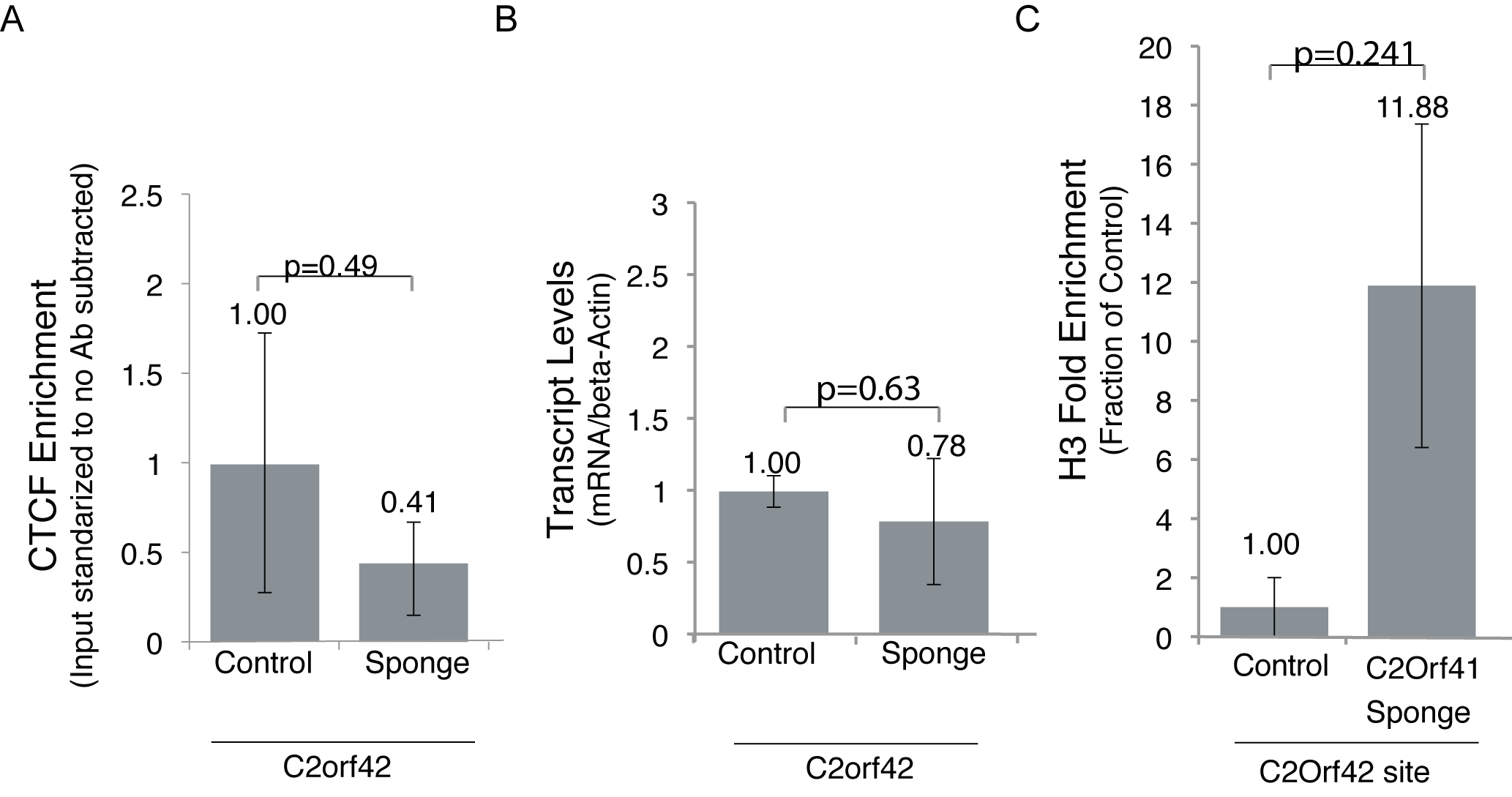

Supplement: Additional file 8 — Figure S6. The effect of the CCCTC-binding factor (CTCF) proximal (cp)-transcription initiation (ti)RNA RNA sponge on CTCF sites proximal to C2orf42. (a) The effects of the C2Orf42 sponge on CTCF localization. (b) The effects of C2Orf41 tiRNA sponges on mRNA expression. Experiments were standardized to pCDNA transfected MCF-7 cells. (c) The effects of the C2Orf41 sponge on histone H3 localization. Samples were analyzed as indicated 72 h post transfection. The averages of triplicate transfected samples are shown with the error bars representative of the standard errors of the means, and P values from paired t tests. (a, c) No antibody values were subtracted from each IP, and the resultant values were standardized to the input for each sample. [file 1756-8935-4-13-S8.PNG]

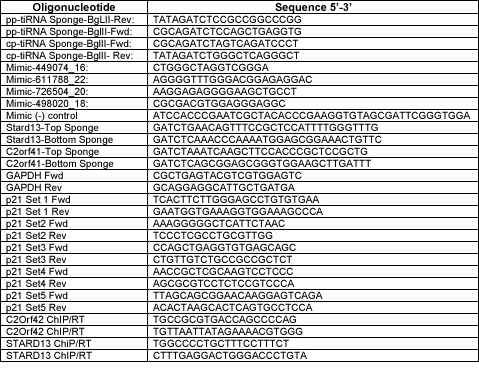

Supplement: Additional file 9 — Table S3. Oligonucleotides used for cloning, quantitative reverse transcription (qRT)-PCR, and chromatin immunoprecipitation (ChIP) analysis. [file 1756-8935-4-13-S9.TIFF]
